# Optical Coherence Tomography Angiography of Early Stage 1a Retinal Hemangioblastoma in Von-Hippel-Lindau

**DOI:** 10.15586/jkcvhl.v8i3.158

**Published:** 2021-09-23

**Authors:** Ananya Goswami, Abhidnya Surve, Pradeep Venkatesh

**Affiliations:** Vitreo-retina, Trauma and Uvea Services, Dr. Rajendra Prasad Center for Ophthalmic Sciences, All India Institute of Medical Sciences, New Delhi, India

**Keywords:** hemangioblastoma, OCTA, retinal capillary hemangioblastoma, VHL

## Abstract

Von-Hippel-Lindau (VHL) syndrome is characterized by focal vasoproliferative tumors of retinal capillaries called retinal capillary hemangioblastomas (RCH). These tumors are initially small and can be easily missed if not looked for carefully. As they grow, these tumors are more demanding to treat and hence the importance of detecting them early and treating them. Herein, we describe and review the optical coherence tomography angiography (OCTA) of the early- stage lesion, which suggested the involvement of superficial and a deeper retinal capillary plexus. In addition, to helping us detect these lesions earlier, OCTA may also help to understand the *in vivo* changes occurring at an earlier phase.

## Introduction

Von-Hippel-Lindau (VHL) syndrome is an exceedingly rare, multi-organ disease with a prevalence of one in 230,000, characterized by the development of focal benign or malignant vasoproliferative tumors (hemangioblastoma). The syndrome occurs because of the mutation in the VHL gene (chromosome 3) and consequent increased expression of vascular endothelial growth factor (VEGF), platelet-derived growth factor (PDGF), erythropoietin (EPO), and transforming growth factor (TGF). Systemic manifestations of VHL include hemangioblastomas in the brain and spinal cord, renal and pancreatic cysts, pheochromocytoma, islet cell tumors, epididymal cystadenomas, endolymphatic sac tumor of the inner ear, and adnexal papillary cystadenoma of probable mesonephric origin (AMPO) of the broad ligament. All the systemic features are manifested in only a few cases with VHL, whereas only 50% of patients display only one systemic feature. Many of these manifestations may occur years after the initial presentation and evolves over a long period (complete penetrance of VHL is seen by 65 years of age) (1).

Retinal capillary hemangioblastoma (RCH) is a clinically visible pathology that develops in 44%–66% of patients, usually in the third decade. These tumors develop denovo from the retinal vascular plexus and progress from an occult angioma to huge angiomas with subsequent complex retinal detachments (2). New lesions develop over a variable time frame and are also detected in the fifth decade of life. In the early stages, the lesions develop away from the fovea, without any visual disturbances. Hence the chances of them getting undetected is high. The various treatment modalities for retinal angioma are laser photocoagulation, cryotherapy, plaque radiotherapy, external beam radiation, proton beam radiation, photodynamic therapy (PDT), trans-pupillary thermotherapy, intraocular injection of anti-VEGF drugs or triamcinolone acetonide (TA), and vitreoretinal surgery (if associated with retinal detachment).

Laser photocoagulation is the most common modality used because of its ease of use, fewer side effects, and results. The different photocoagulation techniques are direct photocoagulation of angioma, treatment of the feeder’s vessel, or a combination of both. Smaller lesions are more amenable to regress with treatment, whereas larger lesions require numerous treatment sessions. However, it is effective in lesions up to 4.5 mm size only and is used for peripheral and juxtapapillary lesions. Apart from laser therapy, trans-scleral cryotherapy is effective for the destruction of these lesions, even in the presence of simultaneous exudation, hemorrhage, or fibrosis. Thus, if we detect these lesions early, they are more amenable to treatment. Therefore, periodic screening is recommended for all patients with VHL (3).

There are various imaging modalities for the diagnosis of retinal angioma. Fluorescein angiography is the most pivotal because of the tumors rich vascularity. On the arterial phase of fluorescein angiography, there is a prominent dilated feeder arteriole along with an adjacent homogenously hyperfluorescent tumor because of a fine capillary network. The venous phase demonstrates an enlarged draining venule. Throughout the late phase of the angiogram, dye leaks into the surrounding tissues (1). While treatment planning, angiography helps to differentiate arteriole from venule. Indocyanine green angiography (ICG) is an adjunct to differentiate choroidal lesions such as a chorioretinal neovascular membrane or choroidal hemangioma from a retinal capillary hemangioma (4). Angioma originates as an inner retinal mass, manifesting as hyper-reflective thickening of the inner retinal layers on optical coherence tomography (OCT) and can displace outer retinal layers with increasing size.

On histopathology, early-stage angiomas show foci of the nodular proliferation of the endothelial cells, abnormal capillary vasculature with later development of reactive gliosis (5, 6). Recently, OCT angiography (OCTA) has become an established modality for noninvasive no-dye assessment of the three (superficial, middle, and deep) vascular layers of the retina (7). In literature, few reports have imaged RCH using OCTA (8–12). Most of these reports are on larger, late-stage angiomas with evident feeder vessels. Table 1 is a summary of these reports and the limitations in angioma description on OCTA. For the first time, this study gives a detailed description of OCTA (obtained using Zeiss Angioplex OCT machine) and its corresponding B-scan of an early stage 1a RCH tumor before the appearance of feeder vessels.

**Table 1:** Studies on optical coherence tomography angiography findings of retinal capillary hemangioblastoma.

Reference	Number of cases	Type of lesion	Stage	Main observations
Lang et al. (8)	10 eyes	2 scars 3 recurrent 3 peripapillary 2 untreated RCH	Untreated RCH - stage B2a and stage B2c	No detailed description of the lesion. Mainly post laser. Suitable to monitor immediate post laser response.
Chou et al. (9)	1 eye	Untreated solitary RCH	Stage B1b. 3 feeder vessels present.	B scan OCT-A shows destruction of all layers of the retina with underlying shadowing. Appears like a hyperreflective mass but individual capillaries not seen.
Sagar et al. (10)	4 eyes	2 juxta-papillary hemangioblastoma 2 Untreated RCH	Case 2 – stage B1b RCH and 1 early stage B1a RCH lesion. Case 3 is same as that described in study number 4.	Case 2- Early RCH OCT-A is not described in detail and not correlated with corresponding B-scan. Emphasized of easier identification of feeder vessels and distinctness of lesions. Relation to various retinal layers not attempted.
Sagar et al. (11)	1 eye	Untreated RCH	Stage B1b. Feeder vessels present. Laser done.	Tumor vascular density shown for one lesion. No detailed description of lesion on OCT-A. No correlation made with corresponding B-scan image.
Chun et al. (12)	2 eyes	2 early lesions – mentioned as subclinical angioma.	Stage B1a (case 1) and stage A0 (case 2) lesions but with no twin vessels.	Early lesions. No detailed description. No twin vessels seen.

RCH: retinal capillary hemangioblastoma; OCT-A: optical coherence tomography angiography.

**Figure 1:**
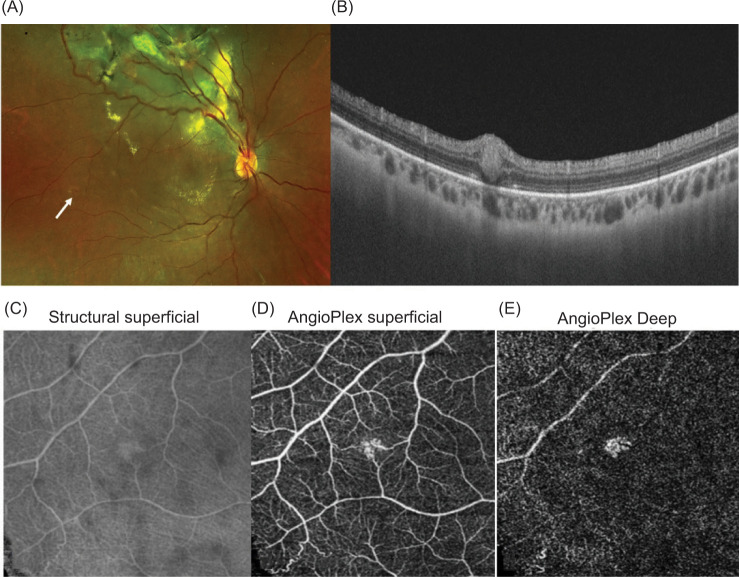
Fundus images shows lasered areas, multiple RCH, and localized retinal detachment. One of these lesions is a barely visible early stage 1a lesion (A- white arrow). B-scan OCT image (B) through this lesion showed the angioma is entirely intraretinal, well defined, and vertically oval. At the lower end, there is basal hypo-reflectivity with absence of an ellipsoid zone and an external limiting membrane. At the upper end, there is a focal hump but with maintained architecture of overlying inner retinal layers. En face structural appearance of the lesion on OCTA (C). An enface image of the superficial retinal layer (D), a focal increase in density and irregularity of the superficial capillary network with no prominent feeder vessels is seen. Adjacent capillary network is normal. An en face image of the deep retinal layers; a relatively more compact, well defined hyperreflective lesion is seen (E). VHL: Von-Hippel-Lindau; RCH: retinal capillary hemangioblastoma; OCTA: optical coherence tomography angiography.

A 33-year-old male diagnosed as VHL presented with decreased visual acuity in both eyes and no other systemic manifestations. Written informed consent was obtained from the patient for investigation, management, and publication purpose. He had a history of surgery in the past for spinal hemangioblastoma. Ocular examination detected multiple angiomas in zone 1 of the right eye and total retinal detachment in the left eye. Left eye vitreoretinal surgery was undertaken while the angiomas in the right eye were treated with direct retinal photocoagulation. The angiomas in the right eye remained stable, and some regressed following laser therapy. The patient underwent routine follow-up examination tri-monthly for early detection of any new angiomas. On a recent follow-up, no diminution of vision was noticed. Fundus examination showed lasered areas, multiple RCH, and localized retinal detachment. One of these lesions was a barely visible early-stage 1a lesion (Figure 1A).

A B-scan OCT lesion image revealed that the angioma was entirely intra-retinal, well defined, and vertically oval. The lower end had a basal hypo-reflectivity with the absence of an ellipsoid zone and an external limiting membrane. The upper end exhibited a focal hump with an intact overlying inner retinal layers (Figure 1B).

On the en face image of superficial retinal layers (Figure 1C-E), a focal increase in density and irregularity of the superficial capillary network with no prominent feeder vessels and the normal adjacent capillary network was seen. En face imaging of deeper retinal layers showed a more compact and well defined lesion. This OCTA of early-stage lesion suggests probable focal involvement of superficial and deep capillary plexus of the retina. In addition to early detection, a further compilation of OCTA findings of early-stage lesions may also help us in understanding in-vivo changes and pathogenesis associated with RCH.
